# Combined therapy of HIFU rectoanal lifting (HIFU-RAL) and sclerotherapy for prolapsed hemorrhoids and rectal mucosal prolapse: A case series

**DOI:** 10.1097/MD.0000000000044368

**Published:** 2025-09-05

**Authors:** Shunsuke Suzuki

**Affiliations:** a Suzuki Proctology-Moriguchi Internal Medicine Clinic, Morioka, Iwate, Japan.

**Keywords:** aluminum potassium sulfate and tannic acid, HIFU rectoanal lifting, high-intensity focused ultrasound, prolapsed hemorrhoid, rectal mucosal prolapse

## Abstract

**Rationale:**

Prolapsed hemorrhoids can impair quality of life due to associated symptoms such as pain. While hemorrhoidectomy is considered the gold standard for treating prolapsed hemorrhoids, this procedure inevitably involves complications such as postoperative pain, bleeding, and delayed recovery. Therefore, there is an increasing need for treatment options that are immediate, effective, and minimally invasive, while also taking into account patients’ physical and social backgrounds, preferences, and values. In this study, we applied a transanal high-intensity focused ultrasound (HIFU) technique, namely HIFU rectal lifting (HIFU-RAL), combined with aluminum potassium sulfate and tannic acid (ALTA) sclerotherapy for treating prolapsed hemorrhoids and assessed its efficacy.

**Patient concerns:**

This case series included 10 symptomatic patients, consisting of 5 men and 5 women. The mean patient age was 67.2 ± 11.0 years, and the median disease duration was 8.0 years (interquartile range: 3.5–38.8 years). All patients complained of prolapse and discomfort that impaired their daily lives.

**Diagnoses:**

Clinical examination identified 8 cases of prolapsed hemorrhoids (Goligher grade III–IV) and 2 cases of rectal mucosal prolapse.

**Interventions:**

HIFU-RAL is a transanal HIFU technique originally developed to treat fecal and urinary incontinence. In this procedure, circumferential ultrasound energy was delivered to the longitudinal muscle of the rectum through the mucosal surface, oral to the dentate line. In ALTA sclerotherapy, the sclerosant was injected into the internal hemorrhoids for prolapsed hemorrhoids and into the submucosal layer of the rectum for rectal mucosal prolapse. The combined efficacy of HIFU-RAL and ALTA sclerotherapy was evaluated over time.

**Outcomes:**

The combined HIFU-RAL procedure and ALTA sclerotherapy were completed in 19.2 ± 6.6 minutes. In all cases, the prolapsed lesions were visibly reduced or resolved immediately after the procedure. During the follow-up period (median: 16.0 months; interquartile range: 10.5–25.8 months), no adverse events or recurrences were observed.

**Lessons:**

The HIFU-RAL procedure combined with ALTA sclerotherapy is a promising, minimally invasive, office-based treatment for prolapsed hemorrhoids. The HIFU-RAL procedure induces contraction of the heated rectal longitudinal muscle, repositioning hemorrhoids to their anatomical site and visibly reducing or eliminating the area of prolapsed lesions.

## 1. Introduction

Hemorrhoidectomy is considered the gold standard for the treatment of prolapsed hemorrhoids; however, it is associated with complications such as pain, bleeding, urinary retention, infection, iatrogenic anal fissure, stenosis, and fecal incontinence.^[1,2]^ Several treatments have been proposed to reduce the risk of complications and recurrence rates.^[3,4]^ In recent years, minimally invasive treatments, such as rubber band ligation, infrared coagulation, and sclerotherapy, which can be performed in an office setting, have been widely applied. Although hemorrhoidectomy remains the standard treatment for Goligher grade III and IV prolapsed hemorrhoids, there is an increasing demand for minimally invasive treatments that can provide immediate relief and can be performed in an office-based setting. Such approaches should consider the patient’s physical and social circumstances, preferences, and values. Nevertheless, the curative efficacy of such treatments remains unclear.

Transanal high-intensity focused ultrasound (HIFU) was originally developed for the treatment of fecal and urinary incontinence, and its clinical efficacy has been previously reported.^[5]^ In patients who received HIFU, shrinkage or resolution of coexisting hemorrhoids, along with elevation of the anal verge, were frequently observed immediately after the procedure. In the present case series, a transanal HIFU technique, named HIFU rectal lifting (HIFU-RAL), for treating prolapsed hemorrhoids was developed. This technique allows for an office-based intervention, combined with aluminum potassium sulfate and tannic acid (ALTA) sclerotherapy. This technique was applied to 8 patients with prolapsed hemorrhoids and 2 patients with rectal mucosal prolapse, and its efficacy was assessed over time.

## 2. Case presentation

### 2.1. Methods

The study involved 8 patients with Goligher grade III and IV prolapsed hemorrhoids and 2 patients with rectal mucosal prolapse following Whitehead surgery (Table 1). Before transanal HIFU, anorectal manometry was performed using the rapid pull-through method with GMMS-100 (Star Medical Co., Ltd., Tokyo, Japan). When thin stools and anal stenosis were observed during digital rectal examination, accompanied by high maximum resting pressure (MRP), insertion of a 23 mm anal cartridge proved difficult. Therefore, lateral internal sphincterotomy (LIS) was performed under local anesthesia before the HIFU-RAL procedure to dilate the anus. Anorectal manometry, LIS, HIFU-RAL procedure, and ALTA sclerotherapy were all carried out with the patients in the left lower lateral recumbent position.

**Table 1:** Characteristics, treatment, and outcomes of patients who underwent high-intensity focused ultrasound rectoanal lifting for prolapsed hemorrhoids and rectal mucosal prolapse.

Case	1	2	3	4	5	6	7	8	9	10
Patient characteristics										
Sex/age (yr)/BMI (kg/m^2^)	Female/72/23	Female/47/22	Female/67/25	Male/61/19	Male/74/22	Male/60/19	Male/59/20	Female/68/19	Male/79/24	Female/85/20
Parity	2	4	2	ー	ー	ー	ー	3	ー	2
Disease duration	40 yr	8 mo	40 yr	4 d	5 yr	8 yr	3 yr	8 yr	50 yr	35 yr
Surgical history (hemorrhoid)	None	None	None	None	None	None	None	Yes	Yes	Yes
Diagnosis (Goligher grade)	**Ⅳ**	**Ⅳ**	**Ⅳ**	**Ⅳ**	**III**	**Ⅳ**	**Ⅳ**	**Ⅳ**	RMP	RMP
Symptom (excluding prolapse)	Anal pain	Anal pain	Anal pain	Anal pain	Bleeding	Discharge	Discharge	Bleeding	Bleeding	Bleeding
	Bleeding	Bleeding	Discharge					Discharge		Discharge
Comorbidities	AF	AF	Hypertension	Constipation	Dyslipidemia	Hypertension		Constipation	Chronic gastritis	AF
	Diabetes	Rectocele		Diarrhea				Diarrhea	Uterine Prolapse	
	Hypertension	Fecal		Hypertension				Dyslipidemia		
MRP/MSP (mm Hg)	56/155	77/108	44/73	71/174	66/152	56/207	86/118	43/188	85/373	45/69
Treatment										
Anesthesia/LIS performed	Spinal/None	Local/Yes	None/None	Local/Yes	None/None	None/None	None/None	None/None	Spinal/Yes	None/None
Cartridge type	Transvaginal	Transanal	Transanal	Transanal	Transanal	Transanal	Transanal	Transanal	Transanal	Transanal
Irradiation count M3.0/D4.5	45/45	45/45	90/90	45/90	90/90	90/90	90/90	90/90	45/90	0/90
ALTA injection dose (mL)	30	27	21	27	27	28	22	24	20	5
Procedure duration (min)	18	20	15	30	21	14	14	17	31	12
Outcomes										
Prolapse recurrence	None	None	None	None	None	None	None	None	None	None
Adverse events	None	None	None	None	None	None	None	None	None	None
Follow-up duration	6 y 9 mo	2 y 2 mo	1 y 7 mo	10 mo	2 y 1 mo	9 mo	6 mo	1 y	4 y 9 mo	1 y 1 mo

AF = Atrial fibrillation, ALTA = aluminum potassium sulfate and tannic acid, BMI = body mass index, LIS = lateral internal sphincterotomy, MRP = maximum resting pressure, MSP = maximum squeeze pressure, RMP = rectal mucosal prolapse.

**Figure 1.**
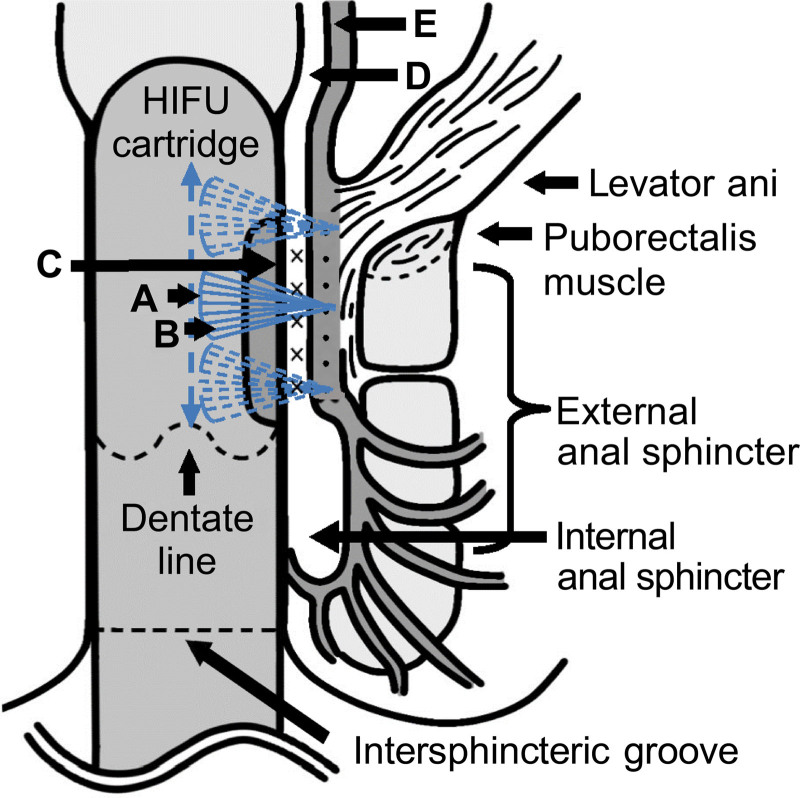
Scheme of delivery of transanal high-intensity focused ultrasound. The diagram depicts the pathway of ultrasound waves emitted from the focused transducer and the irradiation sites. (A) Focused transducer, (B) emitting ultrasound waves, (C) HIFU emitting surface, (D) circular muscle, (E) longitudinal muscle layer. The emitting ultrasound waves (B) generated by the focused transducer (A) converge and penetrate the rectum from the HIFU emitting surface (C). The ultrasound waves are expected to reach the circular muscle (D), located 3 mm deep from the rectal mucosal surface, at the M 3.0 irradiation site (indicated by a cross), and the longitudinal muscle layer (E), located 4.5 mm deep, at the D 4.5 irradiation site (indicated by a filled circle). There is no damage to the tissue between the rectal mucosa and each irradiation site.

After inserting a 23 mm expandable silicone rod into the anus, the cartridge was advanced so that the HIFU emitting surface passed beyond the dentate line, located approximately 1 cm oral to the intersphincteric groove, to reach the rectal muscle layer. This enabled circumferential ultrasound irradiation around the anal canal at 8° intervals, completing 1 to 2 rotations (45 to 90 shots). The irradiation site was estimated based on pain perception during treatment (Fig. 1).

**Figure 2.**
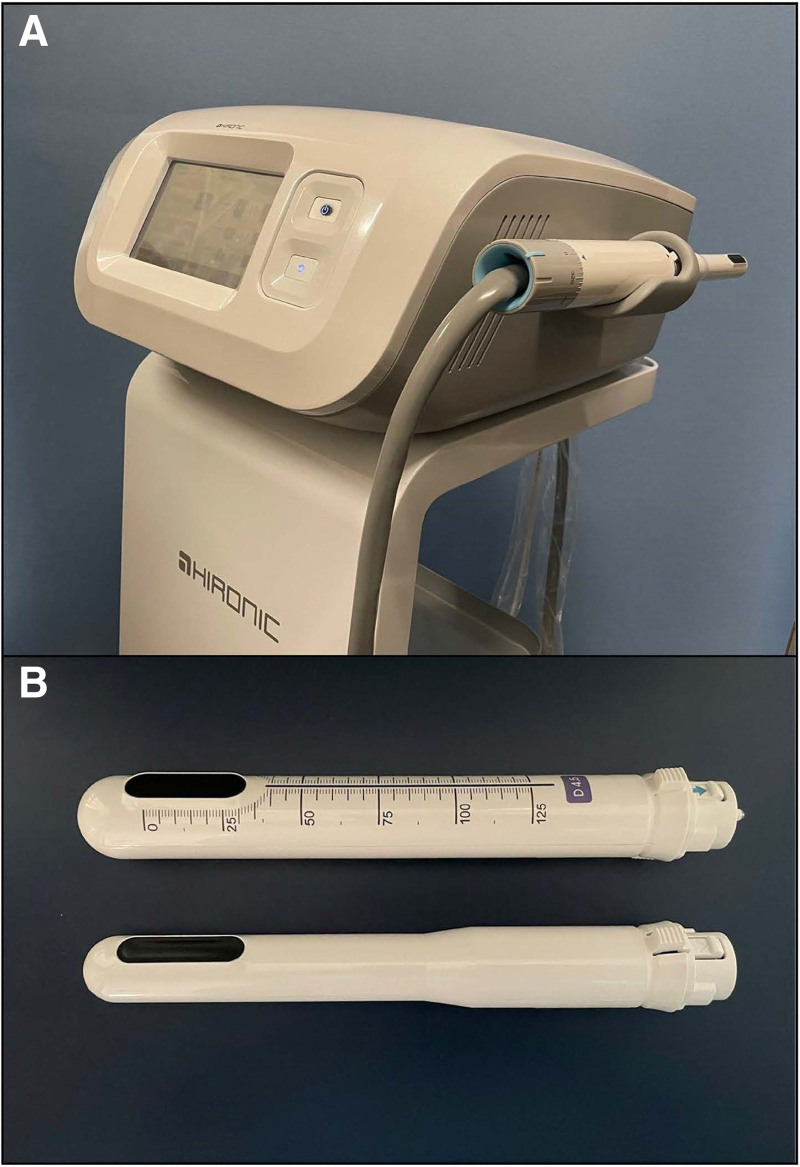
Ultra vera (HIRONIC) device. (A) General view. (B) Upper, transvaginal cartridge (for the vagina) with a diameter of 28 mm. Lower, newly developed transanal cartridge (for the anus) with a diameter of 23 mm.

HIFU was performed using an Ultra Vera™ (Hironic, Co., Ltd., Gyeonggi-do, Republic of Korea), a medical device designed for vaginal rejuvenation and urinary incontinence in female patients (Fig. 2A). In Case 1, the transvaginal cartridge with a diameter of 28 mm provided with the device was used. For Cases 2 to 10, an anal cartridge with a diameter of 23 mm, newly created in collaboration with HIRONIC (Fig. 2B), was used. The irradiation range was 25 mm, with an interval of 1.2 mm. Two types of cartridges were used: D4.5 (frequency 4 MHz, maximum power 2.0J) and M3.0 (frequency 7 MHz, maximum power 1.2J). The irradiation depths were adjusted to 4.5 mm and 3.0 mm from the contact surface, respectively.

For hemorrhoids (Cases 1 to 8), ALTA was injected into 4 specific sites: the submucosa at the superior and inferior poles as well as the central part, and the mucous lamina propria in the central part. For rectal mucosal prolapse (Cases 9 and 10), ALTA was injected into the submucosa at 5 sites along the longitudinal rectal axis, spaced approximately 5 mm apart, using 1 mL per site. In Case 9, injections were administered at 5 sites each on the ventral, dorsal, and lateral (left and right) sides, totaling 20 injection sites. In Case 10, injections were performed at 5 sites only on the ventral side.

For the statistical analysis, data on age, body mass index (BMI), and procedure duration are expressed as mean ± standard deviation (SD). Data on disease duration, ALTA injection dose, and follow-up duration are expressed as the median and interquartile range (IQR). All statistical analyses were performed using EZR (Saitama Medical Center, Jichi Medical University, Saitama, Japan), a graphical user interface for R (The R Foundation for Statistical Computing, Vienna, Austria).

Transanal HIFU was performed after obtaining consent from all patients, and written consent for clinical research was obtained. The study adhered to the ethical guidelines of the Declaration of Helsinki and was reviewed and approved by the Japanese Organization for Safety Assessment of Clinical Research (Approval No. 20240724-02).

### 2.2. Case series

**Figure 3.**
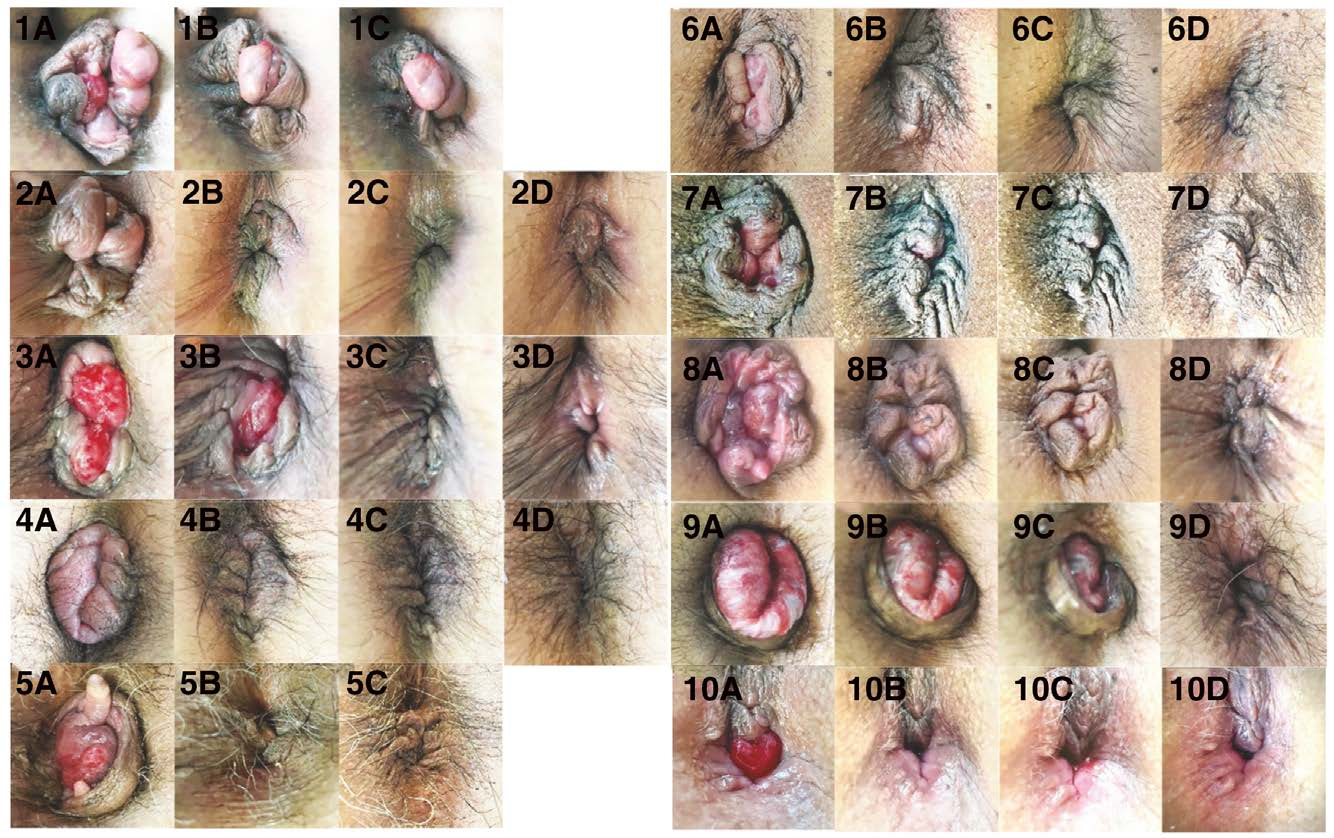
Longitudinal observation before and after HIFU rectoanal lifting and sclerotherapy. Top: Ventral. (1A–1C) Case 1 before, after sclerotherapy (8 min), and after HIFU (18 min), respectively; (2A–2D) Case 2 before, after HIFU (14 min), after sclerotherapy (20 min), and 5 mo later, respectively; (3A–3D) Case 3 before, after HIFU (8 min), after sclerotherapy (15 min), and 4 mo later, respectively; (4A–4D) Case 4 before, after HIFU (13 min), after sclerotherapy (20 min), and 1 mo later, respectively; (5A–5C) Case 5 before, after HIFU (10 min), and 8 mo later, respectively; (6A–6D) Case 6 before, after HIFU (7 min), after sclerotherapy (14 min), and 4 mo later, respectively; (7A–7D) Case 7 before, after HIFU (7 min), after sclerotherapy (14 min), and 3 mo later, respectively; (8A–8D) Case 8 before, after HIFU (11 min), after sclerotherapy (17 min), and 4 mo later, respectively; (9A–9D) Case 9 before, after HIFU (22 min), after sclerotherapy (32 min), and 5.5 hours later, respectively. (10A–10D) Case 10 before, after HIFU (9 min), after sclerotherapy (12 min), and 4 mo later, respectively. The numbers in brackets immediately after indicate the time elapsed since the photograph was taken before HIFU. HIFU = high-intensity focused ultrasound.

Case 1 involved a 72-year-old woman with a history of 2 births, atrial fibrillation, and diabetes (Table 1). The patient had prolapsed hemorrhoids for 40 years and presented to the clinic with difficulty in self-replacement. Circumferential prolapsed hemorrhoids (Goligher grade IV) were observed (Fig. 3-1A). ALTA sclerotherapy was administered under spinal anesthesia to reduce prolapsed hemorrhoids, resulting in immediate shrinkage of the hemorrhoids (Fig. 3-1B). However, as a significant portion of the prolapse remained, HIFU-RAL procedure was subsequently performed, leading to further reduction of the prolapsed area (Fig. 3-1C). The total procedure duration was 18 minutes. As there was no change in the prolapsed area, a hemorrhoidectomy was performed on the affected area after 84 days. No recurrence of hemorrhoids was observed 6 years and 9 months after hemorrhoidectomy.

Case 2 involved a 47-year-old woman with a history of 4 births, atrial fibrillation, and rectocele (Table 1). The patient visited the clinic with an 8-month history of prolapse and difficulty with self-replacement. Circumferential prolapsed hemorrhoids (Goligher grade IV) and anal stenosis due to chronic anal fissure were observed (Fig. 3-2A). As the MRP was high at 77 mm Hg, LIS was performed under local anesthesia, followed by the HIFU-RAL procedure. This resulted in immediate alleviation of the hemorrhoids (Fig. 3-2B). Thereafter, ALTA sclerotherapy was administered, leading to further shrinkage of the hemorrhoids (Fig. 3-2C). The total procedure duration was 20 minutes. Five months after surgery, the prolapsed hemorrhoids had nearly resolved, leaving only skin tags (Fig. 3-2D). No recurrence of hemorrhoids was observed 2 years and 2 months after the therapy.

Case 3 involved a 67-year-old woman with a history of 2 births (Table 1). Immediately after the birth of her second child 40 years ago, the patient experienced prolapsed hemorrhoids, mucus leakage, bleeding, and pain during defecation. Prolapsed hemorrhoids (Goligher grade IV) with mucosal prolapse were observed (Fig. 3-3A). Low values for MRP (44 mm Hg), and maximum squeeze pressure (73 mm Hg) indicated a functional impairment of the anal sphincter muscle. After the HIFU-RAL procedure, performed without anesthesia, most of the internal hemorrhoidal area retracted immediately into the anal canal (Fig. 3-3B). The prolapsed hemorrhoids resolved after ALTA sclerotherapy (Fig. 3-3C). The total procedure duration was 15 minutes. There was no recurrence even after 8 months (Fig. 3-3D) nor after 1 year and 7 months.

Case 4 involved a 61-year-old man with a history of long-term constipation and diarrhea (Table 1). The patient presented to the clinic with painful anal prolapse symptoms that started 4 days prior and difficulty in self-replacement. Complete circumferential edematous external hemorrhoids with anal stenosis were observed (Fig. 3-4A). The MRP (71 mm Hg) was slightly elevated. After LIS under local anesthesia, HIFU-RAL procedure was performed, resulting in almost complete retraction of the prolapsed area into the anal canal (Fig. 3-4B). The prolapse site resolved following ALTA sclerotherapy (Fig. 3-4C). The total procedure duration was 30 minutes. No recurrence was observed at 1 month (Fig. 3-4D) nor at 10 months.

Case 5 involved a 74-year-old man who presented to the clinic with bleeding and prolapse for 5 years, requiring self-replacement (Table 1). The patient requested treatment other than resection. Large anal polyps and prolapsed hemorrhoids (Goligher grade III) were also observed (Fig. 3-5A). After the HIFU-RAL procedure, performed without anesthesia, the prolapse immediately retracted into the anal canal and was resolved (Fig. 3-5B). ALTA sclerotherapy was subsequently administered, with the total procedure duration being 21 minutes. No recurrence was observed at 1 year (Fig. 3-5C) nor at 2 years and 1 month.

Case 6 involved a 60-year-old man who experienced prolapse and soiling of his underwear for 8 years (Table 1). The patient presented to the clinic as it became difficult to self-reduce the prolapse a week prior. Prolapsed hemorrhoids, primarily on the left side (Goligher grade IV), were observed (Fig. 3-6A). The HIFU-RAL procedure was performed without anesthesia, resulting in an immediate reduction of the prolapse (Fig. 3-6B). Subsequently, ALTA sclerotherapy was administered, leaving only a skin tag on the left side (Fig. 3-6C). The total procedure duration was 14 minutes. No recurrence was observed after 4 months (Fig. 3-6D) nor after 9 months.

Case 7 involved a 59-year-old man who had prolapse and soiling of his underwear for 3 years (Table 1). Prolapsed hemorrhoids with mucosal prolapse (Goligher grade IV) were observed (Fig. 3-7A). The MRP was slightly elevated at 86 mm Hg; however, rectal examination revealed no anal stenosis. Owing to the presence of soiling in the underwear, LIS was not performed. The HIFU-RAL procedure, performed without anesthesia, resulted in an immediate reduction of internal hemorrhoids (Fig. 3-7B). Subsequently, ALTA sclerotherapy further reduced the prolapsed hemorrhoids (Fig. 3-7C). The total procedure duration was 14 minutes. No recurrence was noted after 3 months (Fig. 3-7D) nor after 6 months.

Case 8 involved a 68-year-old woman with a history of 4 births (Table 1). The patient presented with difficulty in self-reducing prolapse, accompanied by bleeding and soiling of her underwear during defecation, persisting for 8 years. Circumferential prolapsed hemorrhoids (Goligher grade IV) were observed (Fig. 3-8A). The HIFU-RAL procedure was performed without anesthesia, resulting in an immediate reduction of the hemorrhoids (Fig. 3-8B). This was followed by ALTA sclerotherapy, which further reduced the hemorrhoids (Fig. 3-8C). The total procedure duration was 17 minutes. Postoperative recovery was uneventful, and 1 month later, the patient was able to resume mountain climbing. Four months post-procedure, the prolapsed hemorrhoids had almost completely resolved, leaving only skin tags (Fig. 3-8D). Furthermore, no re-prolapse was observed until 1 year postoperatively.

Case 9 involved a 79-year-old man who underwent surgery for anal prolapse 50 years ago but underwent a second surgery 18 years ago for recurrent anal prolapse (Table 1). Despite 2 previous surgeries, the anal prolapse persisted, and the patient continued to experience bleeding during defecation, leading him to seek further treatment at the clinic. The prolonged prolapse appeared to be a rectal mucosal prolapse after Whitehead surgery, with mucosal organization noted (Fig. 3-9A). Anal stenosis was observed, and MRP (85 mm Hg) was elevated. After LIS, HIFU-RAL procedure was performed under spinal anesthesia, resulting in a confirmed reduction of the prolapse (Fig. 3-9B). Subsequent ALTA sclerotherapy further reduced the size of the prolapsed area (Fig. 3-9C), and the prolapse resolved within 6 hours. The total procedure duration was 32 minutes. No recurrence was observed after 3 years (Fig. 3-9D) nor after 4 years and 9 months.

Case 10 involved an 85-year-old woman with a history of 2 births (Table 1). The patient presented with a 35-year history of prolapse, bleeding, and soiling of her underwear. Rectal mucosal prolapse, primarily on the ventral side, was observed (Fig. 3-10A). The HIFU-RAL procedure, performed without anesthesia, immediately reduced the rectal mucosa (Fig. 3-10B). ALTA sclerotherapy was subsequently administered with similar effects (Fig. 3-10C). The total procedure duration was 12 minutes. No recurrence of prolapse was observed after 4 months (Fig. 3-10D) nor after 1 year and 1 month.

## 3. Discussion

HIFU, initially explored by Lindstrom in 1954 for the treatment of central nervous system disorders, is a minimally invasive technique that involves heating and ablating tissues, eliminating the need for transcutaneous probe insertion into the target site.^[6]^ Presently, HIFU is clinically used for treating prostate cancer and uterine fibroids. Various clinical trials have investigated its application for various tumors, including pancreatic and breast cancers.^[7–11]^ Transanal HIFU was initially developed for the treatment of fecal and urinary incontinence.^[5]^ The present case series describes the application of a combination of the HIFU-RAL procedure and ALTA sclerotherapy for the treatment of 10 symptomatic patients, including 8 with prolapsed hemorrhoids (Goligher grade III–IV) and 2 with rectal mucosal prolapse (5 men and 5 women) (Table 1). The mean age of the patients was 67.2 ± 11.0 years, with a mean BMI of 21.3 ± 2.2 kg/m^2^. The median disease duration was 8.0 years (IQR: 3.5–38.8 years). For Case 1 (the first case), ALTA sclerotherapy was initially planned as the sole treatment. Although it led to a certain reduction in the prolapsed hemorrhoids, this effect remained limited. Therefore, to enhance the outcome, the treatment was supplemented with HIFU, as it was anticipated to promote further shrinkage. This resulted in significantly reduced prolapsed hemorrhoids. Based on this outcome, from Case 2 onward, HIFU was performed first to assess its effect as a stand-alone treatment, followed by ALTA sclerotherapy with an injection dose of 25.5 mL (IQR: 21.3–27.0 mL). The combined HIFU-RAL procedure and ALTA sclerotherapy was completed in 19.2 ± 6.6 minutes. The procedure was well tolerated in all patients, with no interruption or significant changes in vital signs observed during treatment, indicating good adherence and tolerability. In all patients, shrinkage or resolution of the prolapsed area was observed immediately after treatment, and no adverse events or relapses were observed during the follow-up period (median, 16.0 months [IQR, 10.5–25.8]).

HIFU is rapidly gaining clinical acceptance as a treatment that provides noninvasive tissue heating and ablation. Absorption at the target site heats and coagulates the target site for a short period, with minimal effect on the surrounding tissue.^[12]^ The thickness of the internal anal sphincter ranges from 2.2 to 2.7 mm, whereas the thickness of the rectal longitudinal muscle is approximately 1.7 mm.^[13,14]^ This observation indicates that the irradiation site for the M3.0 cartridge in transanal HIFU is positioned near the circular muscle and the internal anal sphincter, located deeper than the anal cushion. In contrast, the D4.5 cartridge targets the rectal longitudinal muscle. In both the M3.0 and D4.5 treatments, the anal cushion of the hemorrhoid was outside the irradiated area. However, a significant reduction in the size of the prolapsed hemorrhoids was visually confirmed. These findings suggest that transanal HIFU may have induced contraction of the circular and rectal longitudinal muscles through thermal coagulation, subsequently leading to an upward displacement of the anal cushion and subsequent hemorrhoidal shrinkage. Sliding or downward displacement of the anal cushion is a contributing factor in the development of hemorrhoids.^[15]^ Notably, the present results suggest that sagging of the rectal wall supporting the anal cushion, due to gravity or increased abdominal pressure, may also be a potential etiological factor for hemorrhoids.

Surgical treatment of hemorrhoids has evolved rapidly over the past few decades. Initially, hemorrhoidectomy was the standard approach, aiming to reduce postoperative pain, time to return to normal, risk of complications, and recurrence rates.^[1]^ More recently, less invasive techniques have been developed that lift the anal cushion without excising hemorrhoids. These techniques include such as the Procedure for Prolapse and Hemorrhoids, Anal Cushion Lifting method, and the Mucopexy-Recto Anal Lifting technique.^[16–19]^ However, these methods involve complex procedures, such as suturing. Therefore, they are typically performed in the operating room, making them challenging to carry out in an office setting. Procedures that can be performed in the clinic include rubber band ligation and sclerotherapy. Rubber band ligation is a highly effective, minimally invasive treatment. Although it is associated with a higher recurrence rate than hemorrhoidectomy, it involves fewer complications, such as postoperative pain, and allows for an earlier return to work. It is widely used as one of the most effective treatments in the United States, the United Kingdom, and other countries.^[20,21]^ However, rubber band ligation is ineffective for extensive prolapsed hemorrhoids. For sclerotherapy, 5% phenol in almond oil is the most commonly used agent. In Japan, ALTA (Zione; J-Dolph Pharmaceutical Co., Ltd., Shiga, Japan) is often employed as a more effective, minimally invasive treatment. ALTA consists primarily of potassium aluminum sulfate and tannic acid. It is an effective agent for managing prolapse and bleeding by inducing hardening and regression of hemorrhoids through fibrosis, which results from granulomatous chronic inflammation caused by potassium aluminum sulfate.^[22]^ ALTA sclerotherapy is particularly useful in cases where surgery should be avoided, such as in older adults or patients on antithrombotic therapy. It is often preferred by both physicians and patients owing to its fewer adverse events.

ALTA monotherapy is effective for Goligher Ⅱ to Ⅲ grade hemorrhoids; however, a high recurrence rate has been reported in Goligher Ⅳ grade cases.^[23–26]^ Therefore, reports have also described combined therapies using ALTA sclerotherapy with distal hemorrhoidectomy or ALTA sclerotherapy with rectal mucopexy.^[27,28]^ The HIFU-RAL method described herein, when combined with ALTA monotherapy, could potentially become a minimally invasive treatment with enhanced efficacy. Notably, HIFU-RAL alone can be performed without anesthesia in an office setting. Depending on patient preferences and subsequent outcomes, additional treatments such as ALTA sclerotherapy, rubber band ligation, infrared coagulation, or surgical options can be selected either concurrently or sequentially.

This study had some limitations. First, it was a single-center retrospective study. Second, the number of patients was limited. Third, the follow-up period was relatively short in several cases, thereby limiting the ability to evaluate long-term efficacy and recurrence. Fourth, although this study primarily relied on the visual assessment of prolapsed hemorrhoids, future studies should incorporate objective evaluation tools, such as symptom severity scales and quality of life questionnaires. Finally, the lack of a comparator arm limits the ability to draw definitive conclusions regarding the relative efficacy and safety of this novel combined therapy. Therefore, large, prospective randomized controlled trials are needed to validate the long-term outcomes and comparative efficacy of this technique.

As society evolves, achieving the Sustainable Development Goals (SDGs) in the medical field becomes increasingly important. Reducing the use of chemicals and minimizing medical waste are key aspects of the SDGs. Combined HIFU-RAL procedure and ALTA sclerotherapy, while not as curative as hemorrhoidectomy, has the potential to contribute to SDGs. It can lead to early shrinkage or resolution of prolapsed hemorrhoids, enhance the physical and mental health of patients, and facilitate a rapid return to work through brief in-office treatment.

## 4. Conclusion

A combined HIFU-RAL procedure and ALTA sclerotherapy was developed and applied for treating prolapsed hemorrhoids. Rectal wall relaxation is believed to contribute to the development of hemorrhoids. Notably, HIFU-induced contraction of the rectal longitudinal muscle helped reposition the hemorrhoids to their anatomical location, resulting in immediate visible shrinkage or resolution after treatment. The HIFU-RAL method holds promise as a minimally invasive, office-based treatment option for prolapsed hemorrhoids. However, further research is needed to determine whether combining HIFU-RAL procedure with ALTA sclerotherapy and other minimally invasive treatments can improve curative outcomes.

## Acknowledgments

I thank Editage for the English language editing.

## Author contributions

**Conceptualization:** Shunsuke Suzuki.

**Data curation:** Shunsuke Suzuki.

**Formal analysis:** Shunsuke Suzuki.

**Funding acquisition:** Shunsuke Suzuki.

**Investigation:** Shunsuke Suzuki.

**Methodology:** Shunsuke Suzuki.

**Project administration:** Shunsuke Suzuki.

**Resources:** Shunsuke Suzuki.

**Software:** Shunsuke Suzuki.

**Supervision:** Shunsuke Suzuki.

**Validation:** Shunsuke Suzuki.

**Visualization:** Shunsuke Suzuki.

**Writing – original draft:** Shunsuke Suzuki.

**Writing – review & editing:** Shunsuke Suzuki.
